# Deformación miocárdica evaluada por ecocardiografía bidimensional en pacientes lúpicos de un hospital nacional

**DOI:** 10.47487/apcyccv.v1i3.66

**Published:** 2020-09-30

**Authors:** Roberto Baltodano Arellano, Kelly Medalid Cupe Chacalcaje, Ciro Barrantes Alarcón, Giovanni Meneses Flores, Gerald Lévano Pachas, Manuel Ugarte Gil

**Affiliations:** 1 Hospital Nacional Guillermo Almenara. Lima, Perú. Hospital Nacional Guillermo Almenara Lima Perú; 2 Universidad Nacional Mayor de San Marcos. Lima, Perú. Universidad Nacional Mayor de San Marcos Universidad Nacional Mayor de San Marcos Lima Peru

**Keywords:** Lupus Eritematoso Sistémico, Ecocardiografía, Cardiomiopatía, Lupus Erythematosus, Systemic, Echocardiography, Cardiomyopathy

## Abstract

**Antecedentes:**

. Los pacientes con lupus eritematoso sistémico (LES) tienen alto riesgo de compromiso cardíaco con elevada mortalidad, por lo que el diagnóstico subclínico podría mejorar su sobrevida. Se ha observado que el strain (deformación) miocárdico longitudinal (SGL) puede ser de utilidad para la evaluación de la función cardíaca en estos pacientes.

**Objetivos.:**

Evaluar la función miocárdica mediante el análisis del strain longitudinal bidimensional (2D), comparar el strain longitudinal entre pacientes con LES y controles, y determinar la correlación con el índice de actividad del LES.

**Material y métodos.:**

Se estudiaron a 44 pacientes con LES (50 ±13 años) y 50 controles (49 ±12 años) pareados por edad y sexo, a quienes se les realizó un ecocardiograma transtorácico bidimensional. Se valoró el strain longitudinal mediante el método de speckle tracking, y la actividad del LES se estimó mediante el Systemic Lupus Erythematous Disease Activity Index (SLEDAI). Se definió como LES activo con una puntuación ≥ 4.

**Resultados.:**

El strain longitudinal global2D fue menor en pacientes con LES que los controles (- 17,3% ± 1,9% vs. - 20%, ± 1,9%; p=0,00). No hubo diferencias en la fracción de eyección de ventrículo izquierdo entre ambos grupos, ni en 2D (p=0,650), ni en 3D (p=0,718). En los pacientes lúpicos, el SLEDAI varió entre 0 y 10, y el 63,8% estuvieron inactivos. Se halló correlaciones negativas significativas entre el puntaje de SLEDAI y la FEVI bidimensional (r de Pearson=-0,372, p=0,017); no se encontró correlación entre el puntaje de SLEDAI y el strain longitudinal global 2D (rho de Spearman=- 0,091 p =0,582).

**Conclusiones.:**

El strain longitudinal global 2D se encontró disminuida en aquellos afectados con LES, lo que sugiere que podría ser una herramienta útil para evaluar la función cardíaca en estos pacientes.

## Abstract (en)

**Background.:**

Patients with systemic lupus erythematosus (SLE) are at high risk of cardiac compromise with high mortality. The subclinical diagnosis may improve their survival. Longitudinal myocardial deformation (strain) has been found to be useful in evaluating cardiac function in these patients.

**Objectives.:**

Our aims were to evaluate myocardial function by analyzing the two-dimensional (2D) global longitudinal strain, to compare the longitudinal strain in SLE patients with controls, and to determine the correlation with SLE activity index.

**Material and Methods.:**

44 patients with SLE (50.0 ± 13 years) and 50 controls (49 ± 12 years) matched by age and sex, underwent transthoracic echocardiogram. Longitudinal strain was assessed using the speckle tracking method and SLE activity was estimated using the Systemic Lupus Erythematous Disease Activity Index (SLEDAI). A score of 4 or more, was defined as active SLE.

**Results.:**

2D global longitudinal strain was lower in patients with SLE than controls (- 17.3% ± 1.9% vs. -20%, ± 1.9% p = 0.00). The left ventricular ejection fraction (LVEF) had no specific differences in both groups in 2D (p = 0.650) or three-dimensional (3D) (p = 0.718). In lupus patients, SLEDAI ranged from 0 to 10, and 63.8% were inactive. Negative correlations were found between the SLEDAI score and 2D LVEF (Pearson's r = -0.372, p = 0.017); no correlations were found between the SLEDAI score and the 2D global longitudinal strain (Spearman's rho = - 0.091 p = 0.582).

**Conclusions:**

2D global longitudinal strain was found to be decreased in the SLE group. This technique might can be a useful tool to assess cardiac function in these patiens.

El lupus eritematoso sistémico (LES) es una enfermedad multisistémica autoinmune que afecta principalmente a las mujeres, con una prevalencia de 51/100 000 habitantes ^1^. El compromiso cardíaco representa una de las principales causas de morbimortalidad en los pacientes con LES, con una prevalencia mayor a 50%; se puede presentar en cualquier fase de la enfermedad y puede afectar todas las estructuras del corazón ^2^. Su presentación clínica es muy variable, principalmente es subclínica y, en algunos casos, puede comprometer la vida del paciente ^3^.

La ecocardiografía transtorácica es una herramienta útil para evaluar el compromiso cardiaco en pacientes con LES; sin embargo, los parámetros ecocardiográficos convencionales, como la fracción de eyección del ventrículo izquierdo (FEVI), no son capaces de detectar la disfunción sistólica subclínica ^4^^,^^5^. Estudios previos muestran el empleo de una nueva técnica ecocardiográfica llamada strain (deformación) miocárdico longitudinal por speckle tracking, que puede detectar de forma más precisa y precoz la disfunción sistólica en estos pacientes, sin embargo, los resultados obtenidos son controversiales ^6^^,^^7^.

El objetivo de este estudio fue evaluar la función miocárdica mediante el análisis del strain longitudinal bidimensional (2D) en pacientes con LES, compararlo con un grupo control y evaluar la correlación del strain longitudinal 2D con el índice de actividad del LES.

## Material y métodos

Estudio prospectivo que incluyó a 44 pacientes con diagnóstico de LES, procedentes del Servicio de Reumatología del Hospital Nacional Guillermo Almenara Irigoyen (HNGAI) en Lima, Perú. Los criterios de inclusión fueron: ausencia de síntomas cardiovasculares y buena ventana ecocardiográfica. Los criterios de exclusión fueron: antecendente de fibrilación auricular, hipertensión arterial, diabetes y la presencia de cardiopatía estructural (FEVI menor a 50%, presencia de prótesis y/o dispositivos cardíacos). Se seleccionaron 50 controles sanos, entre empleados médicos del HNGAI y sus familiares.

Las variables clínicas analizadas en ambos grupos fueron: frecuencia cardíaca (FC), presión arterial sistólica (PAS) y diastólica (PAD) que fueron evaluados antes de realizar la ecocardiografía. Las variables antropométricas analizadas fueron: peso (kg), talla (cm), área de superficie corporal (ASC) y el índice de masa corporal (IMC). Se utilizó una báscula y estadímetro calibrado. Se empleó la fórmula de Du Bois para el cálculo del ASC y se dividió el peso en kg entre el cuadrado de talla en metros para el cálculo del IMC. Los participantes en el estudio firmaron el consentimiento informado antes de ser incluidos.

### Estudio ecocardiográfico

La ecocardiografía se realizó en el área de Imagen del Servicio de Cardiología del HNGAI, con un ecógrafo comercial (GE Vivid E95), equipado con un transductor M5S. El strain longitudinal (2D) se analizó con captura de tres proyecciones apicales (dos, tres y cuatro cámaras) en dos latidos consecutivos. Las imágenes bidimensionales se adquirieron con la misma frecuencia de fotogramas (50-80 cuadros por segundo), se transfirieron a una estación de trabajo y se analizaron off line. Se evaluaron los volúmenes de la aurícula izquierda (AI) y el volumen telediastólico del ventrículo izquierdo, ambos indexados al ASC, y la FEVI mediante el método de Simpson. Se utilizó la técnica del speckle tracking para el análisis del strain longitudinal 2D con el software AFI (EchoPAC BT 12); el software proporcionó valores automáticamente en segmentos y uno global en un bull’s eye (mapa polar) expresados en porcentajes. Los estudios que presentaron más de dos segmentos con tracking inadecuado fueron excluidos. Para el análisis de la fracción de eyección del VI tridimensional (3D), se empleó el transductor matricial 4V con captura de volumen completo con 20-25 fps y se analizó off line.

El análisis ecocardiográfico fue realizado por dos cardiólogos subespecialistas en imagen cardíaca, cuya valoración de la reproductibilidad interobservador e intraobservador fue analizada en un estudio anterior, con una correlación intraclases elevada ^8^.

### Evaluación de la actividad de enfermedad

El índice de actividad clínica de los pacientes con LES fue evaluado antes del estudio ecocardiográfico a través del Systemic Lupus Erythematous Disease Activity Index 2000 (SLEDAI). Se consideró en el estudio como LES activo a una puntuación igual o mayor de 4 puntos ^9^^,^^10^.

### Análisis estadístico

Se empleó la prueba Kolmogorov-Smirnov para determinar la normalidad de las distribuciones de los datos. Las variables que presentaron distribución normal se presentaron como media y desviación estándar y fueron comparadas con la prueba T de Student y r de Pearson; para el resto de variables se emplearon las pruebas no paramétricas Rho de Spearman y U de Mann-Whitney. Mediante el chi cuadrado de Pearson se estableció la asociación de las variables cualitativas. Se utilizó el software estadístico IBM SPSS versión 25, con nivel de significancia estadística de p<0,05.

## Resultados

### Características clínicas de la población

En el estudio se compararon 44 pacientes lúpicos con 50 controles sanos, el 95,5% mujeres. No se encontraron diferencias estadísticamente significativas entre ambos grupos en cuanto a la edad, sexo, presión arterial sistólica, presión arterial diastólica, ni frecuencia cardíaca; por el contrario, se observó que los controles tuvieron significativamente menor peso y, por ende, menor índice de masa corporal (Tabla 1).

**Tabla 1:** Características clínicas de pacientes del estudio

		Pacientes lúpicos (Media ± DE)	Control (Media ± DE)	p
Edad (años)		50,0 ± 13,2	49,0 ± 12,5	0,065†
RF		42/2	42/1	0,570**
Talla (cm)		156,3 ± 5,7	153,6 ± 29,9	0,902†
Peso (kg)		66,3 ± 13,2	65,4 ± 8,8	0,012†
ASC (m2)		1,7 ± 0,1	1,7 ± 0,2	0,106†
IMC (Kg/m2)		27,2 ± 5,6	26,6 ± 3,4	0,022†
PAS (mmHg)		100,0 (100-120) ‡	100,0 (100-120)‡	0,974*
PAD (mmHg)		70,0 (60-80)‡	60,0 (60-80)‡	0,301*
FC (lpm)		71,6 ± 9,5	70,5 ± 7,4	0,510†

DE: desviación estándar. RF: razón de femineidad (femenino/masculino). ASC: área de superficie corporal. IMC: índice de masa corporal. PAS (mmHg): presión arterial sistólica en mmHg. PAD (mmHg): presión arterial diastólica en mmHg. FC (lpm): frecuencia cardíaca en latidos por minuto.

*U de Mann-Whitney. †T de Student para la diferencia de medias. ‡Mediana y rango intercuartil. ** Chi-cuadrado

### Parámetros ecocardiográficos

Se encontró que los controles sanos tuvieron menores volúmenes bidimensionales indexados de ventrículo izquierdo en diástole y de aurícula izquierda. No hubo diferencias significativas en los volúmenes del ventrículo izquierdo indexados 3D, FEVI en 2D ni en modo tridimensional (Tabla 2).

**Tabla 2:** Características ecocardiográficas de pacientes del estudio

		Pacientes lúpicos (Media ± DE)	Control (Media ± DE)	p
Volumen Indexado de Ventrículo Izquierdo 2D (mL/m2)		53,1 ± 11,2	45,1 ± 7,1	0,005†
Volumen Indexado de Ventrículo Izquierdo 3D (mL/m2)		58,0 ± 12,5	55,1 ± 14,1	0,520†
Volumen Indexado de Aurícula Izquierda (mL/m2)		36,2 ± 14,5	31,4 ± 7,7	0,021†
Fracción de Eyección de Ventrículo Izquierdo 2D (%)		59,8 ± 4,1	62,9 ± 4,2	0,650†
Fracción de Eyección de Ventrículo Izquierdo 3D (%)		60,1 ± 3,5	61,9 ± 4,1	0,718†

DE: desviación estándar. *U de Mann-Whitney. †T de Student para la diferencia de medias.

### Evaluación ecocardiográfica de deformación miocárdica

**Figura 1:**
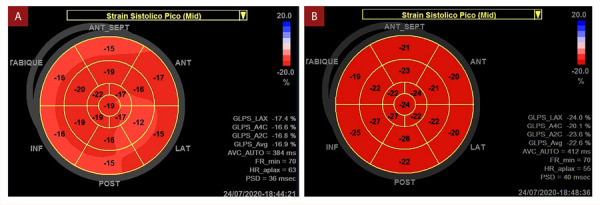
Mapa polar de deformacion miocardica bidimensional A) Caso 1; B) Control de caso 1.

El strain longitudinal global bidimensional 2D fue significativamente menor en pacientes lúpicos con relación al grupo control. Hubo diferencia en los segmentos medios, pero no se encontraron diferencias significativas en los segmentos apicales ni basales (Tabla 3, Figura 1).

**Tabla 3:** Valores de deformación longitudinal global en sujetos lúpicos y sanos

	Control (Media ± DE)	Pacientes lúpicos (Media ± DE)	p
Strain longitudinal global 2D (%)	-20,0 ± 1,9	-17,3 ± 1,9	0,000*
Strain longitudinal basal 2D (%)	-19,9 ± 2,5	-17,4 ± 2,7	0,706†
Strain longitudinal medio 2D (%)	-21,4 ± 2,0	-18,9 ± 2,4	0,045†
Strain longitudinal apical 2D (%)	-23,4 ± 4,1	-21,8 ± 4,4	0,596†

DE: desviación estándar. *U de Mann-Whitney. †T de Student para la diferencia de medias.

En cuanto a los pacientes lúpicos, el SLEDAI varió entre 0 y 10; el 63,6% de los pacientes tuvieron LES inactivo (SLEDAI menor a 4 puntos). Se halló una correlación negativa significativa entre el puntaje de SLEDAI y la FEVI 2D (r de Pearson=-0,372, p=0,017), pero no con la FEVI 3D (rho de Spearman=-0,284, p=0,084). No hubo correlación significativa entre el puntaje de SLEDAI y el strain longitudinal global 2D (rho de Spearman=-0,091, p=0,582). Aunque hubo tendencia a una correlación negativa (rho de Spearman=-0,310, p=,055) entre el puntaje de SLEDAI y el strain longitudinal basal 2D, no se encontró con el strain longitudinal apical 2D (rho de Spearman=- 0,007, p=0,965) ni con el strain longitudinal medio 2D (rho de Spearman=- 0,113, p=0,494).

## Discusión

Los avances en el tratamiento del LES han mejorado la sobrevida de los pacientes lúpicos; sin embargo, aún persiste una elevada morbimortalidad por la afección cardíaca ^11^^-^^14^. Los estudios reportan entre 40 - 50% de afección cardíaca en las autopsias de pacientes lúpicos, pero solo se reporta entre el 7 - 10% de casos en vida ^15^^,^^16^. El análisis de la deformación miocárdica a través del método de speckle tracking es una técnica semiautomática de uso creciente en los últimos años que ha mostrado ser útil en diferentes escenarios clínicos como la enfermedad reumatológica, sobre todo en la afección cardíaca subclínica ^17^^-^^19^.

Este estudio presenta una muestra de pacientes lúpicos de un único centro hospitalario, compuesto, en su mayoría, por mujeres asintomáticas cardiovasculares, en quienes se analizó el strain longitudinal 2D y se las comparó con una muestra de controles sanos, cuyos resultados mostraron un promedio de strain longitudinal global 2D significativamente menor en los pacientes lúpicos que en los controles, sin encontrar diferencias significativas en parámetros convencionales como la FEVI 2D y 3D, lo que podría indicar una afección cardíaca incipiente en los pacientes lúpicos, sin afectar aún la FEVI; con los cambios iniciales, como el aumento de volumen de AI y VI, que sí muestran diferencias significativas entre ambos grupos, el diagnóstico precoz de la afección cardíaca puede favorecer el tratamiento adecuado y oportuno en pacientes lúpicos ^20^^,^^21^.

Un metaanálisis reciente mostró la comparación del estudio del strain longitudinal global en 457 pacientes lúpicos y controles sanos; en ellos, la prevalencia de LES fue mayor en mujeres, y el promedio del strain longitudinal global fue menor en pacientes lúpicos. Sin embargo, la mayor limitación de los estudios incluidos fue la heterogeneidad en los criterios de inclusión de los pacientes; aun así se sugiere al strain longitudinal global como una herramienta útil en el diagnóstico precoz de la afección cardíaca ^4^. En otro estudio reciente, Saad et al., encontraron en 37 mujeres lúpicas un promedio de strain longitudinal global 3D menor que los controles y esta disminución fue aun más acentuada en las pacientes con LES activo ^5^.

A diferencia de los estudios mencionados, encontramos que menos del 40% de los pacientes lúpicos tuvieron LES activo, y no se encontró correlación entre el puntaje del SLEDAI y el valor del strain longitudinal global 2D, esto probablemente se deba a que nuestra muestra tenía pacientes con menor puntaje de SLEDAI, lo que difiere de los estudios anteriores ^4^^,^^5^.

La deformación miocárdica tridimensional (strain 3D) es una técnica de imagen avanzada que se basa en la adquisión de una imagen volúmetrica (3D), que permite un análisis de múltiples parámetros de deformación con una sola adquisión. A pesar de los grandes avances tecnológicos sigue siendo una técnica joven que tiene limitaciones en la adquisición por ser dependiente de una buena ventana ecocardiográfica; además, se requiere que el paciente contenga la respiración, un ritmo regular, mayor demora de adquisión y dependen del equipo utilizado en más medida que el strain 2D. Por lo tanto, consideramos realizar el análisis bidimensional por ser más accesible, factible y de utilidad clínica en nuestra población.Creemos que el strain 3D es una tecnología de gran utilidad que deberá ser validada en futuras investigaciones ^22^.

## Limitaciones

Los hallazgos son aplicables a pacientes examinados con el equipo utilizado en este estudio. Los resultados no se pueden extrapolar a otra población.

## Conclusiones

Nuestros resultados muestran que el strain longitudinal global 2D se encontró disminuido en pacientes con LES, lo que sugiere que podría ser una herramienta útil para la evaluar la función cardíaca en estos pacientes con afección cardíaca subclínica. Consideramos importante determinar, en el seguimiento de estos pacientes, si las diferencias encontradas tienen algún valor predictivo en la presencia de eventos cardiovasculares.
